# Oncolytic viruses in melanoma

**DOI:** 10.31083/j.fbl2702063

**Published:** 2022-02-14

**Authors:** Camille Robinson, Maria M Xu, Smita K Nair, Georgia M Beasley, Kristen E Rhodin

**Affiliations:** 1 School of Medicine, Duke University, Durham, NC 27710, USA; 2 Department of Surgery, Duke University Medical Center, Durham, NC 27710, USA

**Keywords:** melanoma, in transit melanoma, intralesional therapy, oncolytic viruses

## Abstract

Malignant melanoma recurrence remains heterogeneous in presentation, ranging from locoregional disease (i.e., local recurrence, satellites, in transit disease) to distant dermal and visceral metastases. This diverse spectrum of disease requires a personalized approach to management and has resulted in the development of both local (e.g., surgery, radiation, intralesional injection) and systemic (intravenous or oral) treatment strategies. Intralesional agents such as oncolytic viruses may also evoke local immune stimulation to induce and enhance the antitumor immune response. Further, it is hypothesized that these oncolytic viruses may convert immunologically “cold” tumors to more reactive “hot” tumor microenvironments and thereby overcome anti-PD-1 therapy resistance. Currently, talimogene laherparepvec (T-VEC), a modified herpes virus, is FDA-approved in this population, with many other oncolytic viruses under investigation in both preclinical and trial settings. Herein, we detail the scientific rationale, current landscape, and future directions of oncolytic viruses in melanoma.

## 1. Introduction

**Fig. 1.**
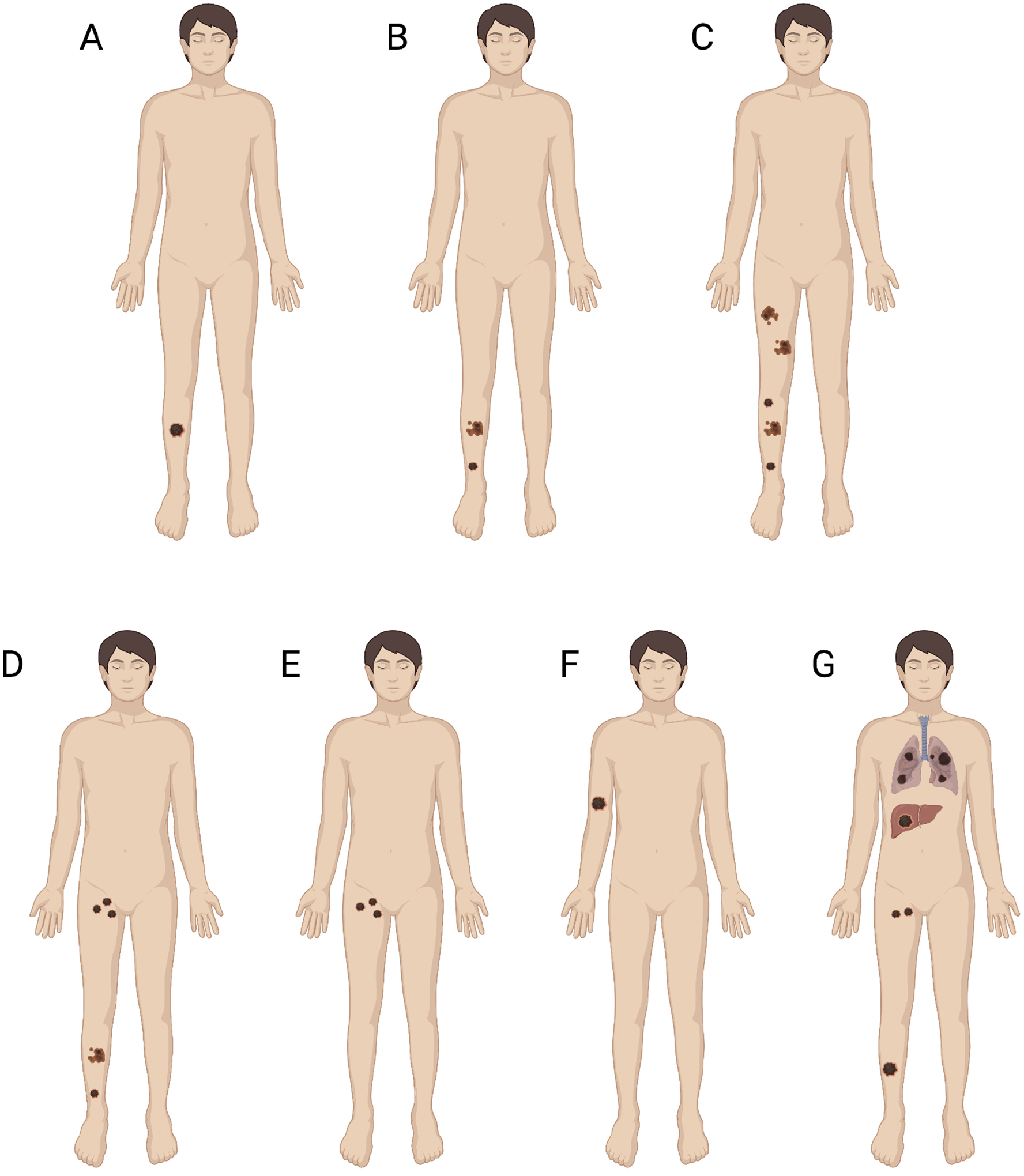
Spectrum of Recurrent and Advanced Melanoma. (A) Local Recurrence. (B) Satellite and In Transit Lesions. (C) Extensive In Transit Disease. (D) In Transit and Nodal Disease. (E) Nodal Disease. (F) Distant Dermal Metastasis. (G) Distant Visceral or Intracranial Metastasis. Figure created with BioRender.com.

Cutaneous malignant melanoma remains the most lethal form of skin cancer. While many early melanomas are curable with surgical resection alone, recurrence is not uncommon and occurs in 8–40% of patients [1–3]. Recurrence is not uniform, but rather highly heterogeneous ranging from locoregional disease to distant metastases (Fig. 1). Locoregional disease includes surgical site recurrences, nodal metastases, as well as satellite and in transit (IT) lesions. IT melanoma is a unique pattern of disease with involvement of dermal and subdermal lymphatics anywhere from the primary site to its draining lymph node basin. Even in the setting of adequate primary resection, IT melanoma recurrence occurs in 4–10% of patients within a median time of 18 months following surgery [4,5]. Beyond the regional lymph node basin, patients may develop dermal, visceral, or intracranial metastases. These can occur in isolation or patients can have a combination of locoregional and distant disease. Regardless, the development of recurrent or metastatic melanoma, including IT and dermal metastases, has significant implications on survival with 66% and 27% five-year survival for regional and distant disease, respectively [6].

In transit disease and dermal metastases pose similar challenges to treating physicians. IT lesions range in size and number, with some patients experiencing significant morbidity from pain, itching, bleeding, or infection. Similarly, patients with dermal metastases may have concurrent visceral or intracranial metastases. Traditional treatment strategies included surgical metastasectomy and regional (limb-infused) or systemic chemotherapy, though have broadened within the past decade to include more effective systemic therapies (immune checkpoint inhibitors [ICI] and targeted therapy) in addition to intralesional immunotherapy. The variable disease burden in IT disease does not always allow for excision and in those with isolated lesions, patients remain at high-risk of recurrence without additional treatment. Alternatively, isolated limb infusion and hyperthermic isolated limb perfusion include administration of high-dose chemotherapy to the involved limb while limiting systemic exposure. While these regional strategies can have a complete response rate of 30–40%, up to 85% of patients experience recurrence within three years [4,7,8].

Historically, systemic chemotherapy has had limited utility in metastatic melanoma; however, the advent of modern immune therapies (ICI—anti-PD-1 and anti-CTLA-4) and targeted therapy (BRAF/MEK inhibitors) has revolutionized outcomes in this population [9,10]. While these agents have high utility, their use can be limited by severe toxicities, cost, and resistance [11]. Alternatively, intralesional therapies are injected locally within tumor deposits. The accessibility of IT lesions and dermal metastases lends well to this strategy and is generally well tolerated. Intralesional therapies include oncolytic viruses, proinflammatory cytokines, innate immune agonists, and vaccines. In brief, these therapies work through local anti-tumor effects with the aim of recruiting immune infiltrates into the tumor and propagating the systemic antitumor immune response. These agents may directly kill cancer cells and or alter the tumor microenvironment such that modern ICI can be more effective. The oncolytic virus talimogene laherparepvec (T-VEC), was approved by the United States Food and Drug Administration (FDA) in 2015 in this setting and several others are in preclinical development or under active investigation in clinical trials. This review highlights the scientific rationale of oncolytic viruses in melanoma, their current clinical uses, ongoing development, and challenges to clinical trial design.

## 2. Scientific rationale

Broadly, intralesional immune therapies are designed to deliver immunostimulatory agents directly to the tumor microenvironment that may then boost both the local and systemic antitumor immune response. This phenomenon has been likened to the tumor serving as its own vaccine, a concept derived from the observation that durable tumor responses reflect an interplay of immunostimulatory and suppression of regulatory responses [12]. This concept has been illustrated with immunogenic cell death being a key component of effective antitumor treatments such as chemotherapy and radiation [13]. The success of immune checkpoint blockade in prolonging survival for any cancer types has conversely illustrated the effectiveness of suppression of regulatory responses [12]. The side effect profile of systemic and locally targeted therapies such as chemotherapy, radiation, and immune checkpoint blockade highlight the delicate balance that both boosting inflammation and regulation of its suppression play in effective antitumor responses that translate into improved morbidity, mortality, and disease-free survival.

Intralesional immune therapies have been developed to mimic the systemic immunogenicity of vaccines [14]. Local injection of immunostimulants such as cytokines and coated nanoparticles have been shown to generate polyclonal immune stimulation that then translates into lasting adaptive immunity [15]. Contrary to personalized cancer vaccines which target specific tumor antigens or mutations within tumor antigens, administration of these agents allows a patient’s immune system to self-select the most immunogenic antigen within a given lesion. Many of these patients have multiple lesions and mutational burden is certainly variable among metastases [16]. Injection of multiple lesions may combat the heterogeneity of metastases and create a more robust antitumor immune response with durable memory. Abscopal responses, or tumor responses occurring in noninjected lesions, have also been demonstrated, although the systemic efficacy of these agents is debated [17,18].

**Fig. 2.**
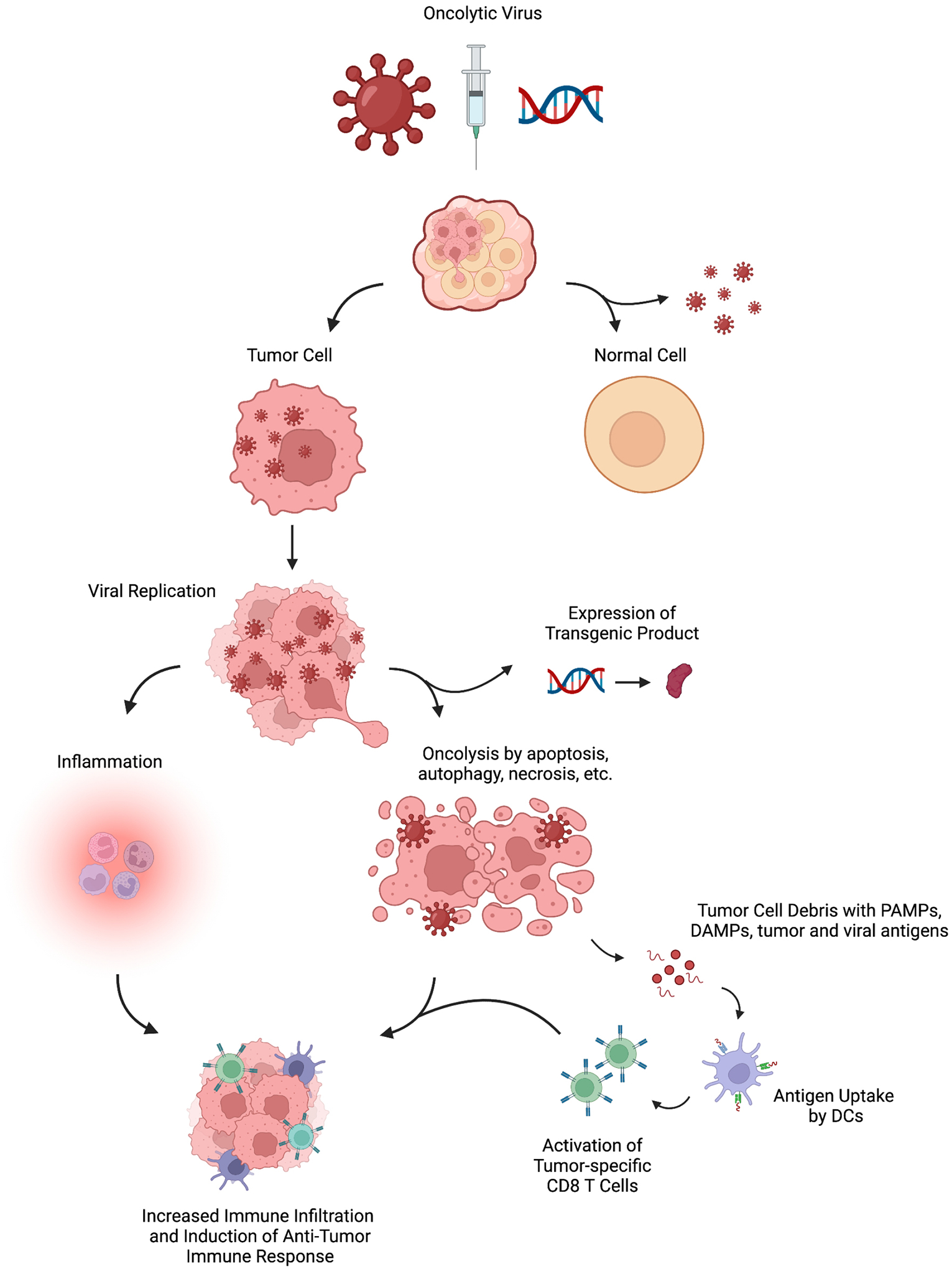
Mechanisms of Oncolytic Viruses highlighting their selective replication within tumor cells, oncolysis, increased inflammation and immune cell infiltration. Figure created with BioRender.com.

Beyond these broad mechanisms, oncolytic viruses exhibit selective replication within tumor cells and take advantage of their direct cytotoxicity in addition to stimulating anti-tumor immunity (Fig. 2). Tumor cells often have a deficient response to the stress of viral infection compared to their healthy counterparts, which allows for continued replication [19]. The cytotoxicity of oncolytic viruses is likely dependent on this selective viral replication within tumor cells, while sparing normal cells, and subsequent induction of apoptosis, autophagy, or necrosis [19–21]. The mechanisms by which each virus accomplishes this vary and are incompletely understood. Some of the described mechanisms involve induction of apoptosis by early cleavage of caspases, surface exposure of calreticulin, or autophagy following infection as a prosurvival strategy [19–21]. In addition, the local inflammatory response created by the administration of the virus into the tumor and resulting cell death has the potential to recruit and activate additional immune cells [19]. This strategy may in fact be able to convert immunologically “cold” tumors, or those with low levels of proinflammatory cytokines and CD8^+^ T cell infiltration at baseline, to “hot” tumors [22–24]. Ribas and colleagues, in a phase I study, have demonstrated such changes in the tumor microenvironment with increases in CD8^+^ T cells after T-VEC [25]. The ability to transform the tumor immune environment has significant implications for combined utilization with ICI. The potential synergy of these treatment strategies is of active interest, particularly if there is a role in overcoming resistance to ICI. In this setting, tumor responses occurring in noninjected lesions after combination therapy highlight the anenestic response, a pattern distinct from the previously described abscopal response. Altogether, the scientific rationale underlying oncolytic viruses is certainly exciting and provides motivation for their clinical study in melanoma.

## 3. Current clinical uses

At this time, talimogene laherparepvec (T-VEC) remains the only oncolytic virus approved by the United States Food and Drug Administration (FDA). Initially named OncoVEXGM-CSF in its early development, T-VEC is designed to replicate within tumor cells causing lysis of these cells while establishing antitumor immunity. In brief, this virus is a modified HSV-1 strain in which the neurovirulence gene, ICP34.5, and the inhibitor of antigen presentation, ICP47, have been deleted [19,26]. The deletion of ICP34.5 prevents infection of neurons while increasing cancer cell selectivity [19,27]. Alternatively, deletion of ICP47 allows for viral antigen presentation and early activation of the US11 promoter which prevents tumor cells from undergoing abortive apoptosis after infection [19,28]. Further, T-VEC is engineered to include the gene for GM-CSF to improve the antitumor immune response [19].

Following promising preclinical studies, a phase I clinical trial conducted by Hu *et al*. [29], first analyzed the safety profile and biological activity of OncoVEXGM-CSF (T-VEC). Patients with refractory cutaneous or subcutaneous metastases from various types of cancer, including malignant melanoma, received an intratumoral injection of the virus [29]. Overall, the virus was well tolerated, with grade 1 pyrexia, constitutional symptoms, and local inflammation as the most common side effects. Importantly, this trial informed dosing regimen development, as results demonstrated that a single dose of 10(6) pfu/mL followed by multiple doses of 10(8) pfu/mL was a viable regimen that could be delivered every 2–3 weeks with decreased risk of local reactions [29]. Clinically, there were no complete or partial responses as a result of the OncoVEXGM-CSF virus, although three patients had stable disease. Many patients had either flattened lesions or no further progression. Interestingly, inflammation and necrosis were found in 14/19 biopsies taken after injection [29]. Further, necrosis was isolated to HSV-infected tumor and not found in healthy tissue [29].

Expanding on early results, the phase II clinical trial conducted by Senzer *et al*. [30], was designed to investigate the efficacy of T-VEC. The investigators studied overall response rate, categorized into partial and complete response, and safety profile in patients with unresectable metastatic melanoma. Patients were given an initial dose and secondary dose 3 weeks later with additional doses completed every 2 weeks for up to 24 treatments [30]. Fifty patients enrolled and received a median of 6 injection sets [30]. They reported an objective response rate of 26% (N = 8/50 complete response; N = 5/50 partial response) and one-year survival of 58% [28]. Notably, 92% of patient responses in both injected and noninjected lesions were maintained for 7 to 31 months suggesting systemic efficacy and durability of this virus [30].

The OPTiM multicenter phase III trial conducted by Andtbacka *et al*. [17] randomized 436 patients with unresected stage IIIB, IIIC, and IV melanoma to either intratumoral injection with T-VEC or subcutaneous recombinant GM-CSF. Both durable response rate (DRR) and overall response rate (ORR) were greater in the T-VEC arm compared to GM-CSF (DRR 16.3 vs 2.1%, *p* < 0.001; ORR 26.4 vs 5.7%, *p* < 0.001) [17]. In addition, median survival was prolonged in patients receiving T-VEC (23.3 vs 18.9 months, *p* = 0.051). These results led to the FDA’s approval of T-VEC for intralesional treatment of unresectable stage III and IV melanoma in 2015. Importantly, analysis at long-term follow-up (median 49 months) maintains these earlier findings [31]. Further analysis suggests that both early metastatic melanoma and lower tumor burden are independent predictors in the achievement of a complete response. The median time to achieve complete response for patients in the T-VEC arm was 8.6 months and complete response was associated with improved overall survival [31].

Despite the promising results highlighted above, it is important to note that T-VEC has not been shown to improve overall survival when used alone. Therefore, the use of T-VEC in combination with systemic therapy, specifically ICI, is of high interest. A randomized phase II trial conducted by Chesney *et al*. [32], evaluated the combination of T-VEC and ipilimumab versus ipilimumab alone. Combination therapy resulted in higher overall response rates (OR 2.9; 95% CI 1.5 to 5.5; *p* = 0.002) when compared to ipilimumab monotherapy [32]. There was also evidence of systemic antitumor response, as the investigators found that clinically, these responses targeted both injected and noninjected tumors. Additionally, there were high rates of complete reduction in visceral tumor burden even though these tumors were not injected [32]. Alternatively, investigation of T-VEC with PD-1 therapy has been completed. In a phase Ib trial, Ribas *et al*. [25] describe promising response rates (ORR 62%, complete response rate 33%) in patients with advanced melanoma who received combination T-VEC and pembrolizumab [25]. More recently, Masterkey-265, a phase III randomized, double-blind trial was conducted to assess the efficacy of T-VEC and pembrolizumab in combination compared to placebo-pembrolizumab for unresectable melanoma. Although the median progression-free survival (PFS) was 14.3 months for T-VEC and pembrolizumab combination therapy versus 8.5 months for placebo-pembrolizumab, this did not meet statistical significance and there was no significant improvement in 1- and 2-year PFS (HR 0.86; 95% CI 0.71 to 1.04; *p* = 0.13) [33]. Similarly, no significant overall survival benefit was detected with combination therapy. Further analysis and discussion of these results is eagerly anticipated in the final manuscript.

Altogether, these findings suggest that the efficacy of T-VEC may not compete with the robust systemic antitumor immune response provided by ICI. However, in the current therapeutic setting there are undoubtedly patient populations that will benefit and for whom T-VEC should be considered. These populations include those with poor functional status, who cannot tolerate or are refractory to ICI as well as patients with advanced or unresectable metastatic melanoma who may seek reduction in disease burden for palliative purposes [34]. While current systemic therapies offer the potential for previously unseen efficacy, not all patients experience disease response and some develop resistance. The heterogeneity of advanced melanoma and its response to therapy provide further motivation for the utilization of T-VEC and development of other oncolytic viruses.

## 4. Oncolytic viruses in development

### 4.1 ONCOS-102 adenovirus

ONCOS-102 is a modified adenovirus that expresses GM-CSF and binds the desmoglein 2 receptor often expressed on tumor cells [35,36]. Once within the tumor cell, deletion in the Rb binding site of the E1A gene restricts replication to cells with p16-Rb pathway defects, which is common in most cancers [35,36]. Importantly, GM-CSF production remains isolated within the tumor microenvironment while systemic exposure remains limited [35]. Resultant recruitment of natural killer and cytotoxic T cells into the tumor has been demonstrated in both animal models and human studies [35]. Preclinical studies conducted by Kuryk *et al*. [35] demonstrated notable anti-tumor effects of ONCOS-102 and pembrolizumab combination therapy. In humanized mice bearing A2058 tumors, investigators found that ONCOS-102 treatment alone resulted in 51.5% tumor volume reduction and in combination with pembrolizumab resulted in approximately 60% tumor volume reduction.

In a phase I trial, Ranki *et al*. [37] demonstrated safety and efficacy of ONCOS-102. Of the 12 patients enrolled, 4 had stable disease at 3 months. Interestingly, post-treatment tumor infiltration by CD8^+^ T cells for 11 out of the 12 patients was observed with a median fold change of 4.0 from baseline [37]. There was also a positive correlation between infiltrating CD8^+^ lymphocytes and overall survival. Together these findings suggest the ability of ONCOS-102 to regulate tumor microenvironment by recruiting immune cells with cytotoxic properties. An additional phase I trial (NCT03003676) studying the combination of ONCOS-102 with pembrolizumab has recently completed and announced promising early results with an ORR of 33% in anti-PD-1 refractory melanoma [38]. With these novel findings, the FDA has granted a fast-track designation to ONCOS-102 for patients with anti-PD-1 refractory advanced melanoma [38].

### 4.2 Coxsackievirus A21

Coxsackievirus A21 (CVA21, CAVATAK, V937) is an unaltered RNA virus that can selectively infect tumor cells by entry through intercellular adhesion molecule-1 (ICAM-1) and decay-accelerating factor (DAF), which are both overexpressed in metastatic melanoma and other malignancies [34,39,40]. Advantages of CVA21 include that it does not require genetic modification for safety and the natural cell lysis observed with infection [19]. The release of damage-associated molecular patterns (DAMPs), such as calreticulin, following tumor cell lysis promotes further immune cell infiltration and release of type 1 IFNs [19].

The phase II CALM trial evaluated immune-related PFS (irPFS) after injection of CVA21 in patients with unresectable stage IIIC to IVM1c melanoma. 21 of the 57 (36.8%) patients enrolled achieved irPFS at 6 months with an ORR of 28.1% [39,40]. There was a≥ 6 months DRR of 19.3% and significantly, the median time to response was 2.8 months [39]. The phase I MITCI trial (NCT02307149) investigated CVA21 in combination with ipilimumab in patients with previously treated or untreated unresectable melanoma. At interim analysis, 23 patients with stage IIIB/C to IVM1c melanoma were enrolled and the combination therapy was well tolerated. Of the 18 evaluable patients, 9 (50%) had an overall response including both ICI-naïve and treated patients [41]. Although further studies are ongoing, combination CVA21 and ipilimumab is a promising treatment option for patients with anti-PD-1 refractory disease.

### 4.3 Poliovirus (PVSRIPO)

PVSRIPO is a live-attenuated, recombinant poliovirus type 1 of interest for treatment of advanced melanoma, among other malignancies. Its neurovirulence is limited by the inclusion of the internal ribosome entry site of human rhinovirus type 2 [34]. Selective tumor cell infection occurs through CD155, the poliovirus receptor, which is up-regulated on many malignancies including melanoma [42]. Cytopathogenic damage to tumor cells can occur. In addition, PVSRIPO infects antigen-presenting cells resulting in their activation and sustained type I and III interferon (IFN) responses within the tumor microenvironment [43–45]. Altogether, this leads to increased immune cell infiltration and recruitment of T lymphocytes [45]. In the phase I trial, Beasley *et al*. [46] confirm the safety of PVSRIPO in patients with unresectable stage IIIB/C and limited stage IV melanoma. The most common adverse effect (60%) was localized pruritus, and no grade 3 or higher adverse effects were observed [46]. Although 33% (4/12) of patients achieved an objective response, 67% did not have any clinical benefit [46]. Overall, these results demonstrate promise and PVSRIPO is now under investigation in combination with anti-PD-1 therapy (NCT04577807).

### 4.4 Other viruses in development

Vesicular stomatitis virus (VSV) has demonstrated tropism to malignancies with IFN signaling defects, which is common in melanoma [47]. Previous study of human melanoma samples has demonstrated widespread infection and lysis of melanoma tumor cells *in vitro* compared to melanocytes [47]. An ongoing phase I trial (NCT03865212) is investigating a modified VSV that includes genes encoding human IFN beta, which may protect healthy cells from the virus, and TYRP1, an antigen expressed in melanocytes.

Preclinical study of the maraba virus demonstrates oncolytic activity against many cell lines of human, canine and murine origin. The maraba virus utilizes the low-density lipoprotein receptor (LDLR) for its entry into tumor cells to impose direct cytotoxicity [48]. Its efficacy has led to the development of cancer vaccinations, the MG1-infected cancer cell vaccine and MG1-based prime-boost vaccine, both of which work collectively to induce cell death, local release of DAMPs, produce a type I IFN response and reinforce innate and acquired antitumor responses [48]. While it has not been trialed in melanoma, early-phase clinical trials have begun in other malignancies (NCT02285816; NCT02879760).

Further, other oncolytic herpesviruses are in development. HF10, an attenuated herpes simplex virus, has been studied in breast, oral, head and neck, and pancreatic cancer with confirmed oncolytic activity [49]. In a preclinical study conducted by Esaki *et al*. [50], HF10 resulted in tumor cell death without harming normal cells, increased T cell infiltration and necrosis, and durable antitumor immunity as demonstrated by rejection of secondary tumor challenge. RP1 (Replimune), a HSV strain that encodes GALVGP R and GM-CSF, is being studied alone and in combination with nivolumab in patients with melanoma and non-melanoma skin cancers (NCT03767348). On preliminary analysis, objective treatment responses have been observed, and 3/4 anti-PD-1 refractory patients have been responding to treatment [51]. Additionally, tumor biopsies have confirmed recruitment of CD8^+^ T cells and increased PD-L1 expression [51]. Similarly, OrienX010 is a modified HSV-1 strain engineered to express GM-CSF and is being studied for metastatic melanoma to the liver. Evidence from the phase I trial suggested a potential benefit, as overall response rates were 8.3% and the disease control rate was 41.7%, with one patient having partial response and four having stable disease [52]. A phase II trial is currently ongoing (NCT04200040).

## 5. Trial design and future directions

Ongoing development and investigation of oncolytic viruses alone or as combination therapy requires creative trial development and interpretation. In comparison to the highly standardized and protocolized trial designs for systemic therapies, investigation of intralesional agents has many unique challenges. While the target population may have uniform stage or designation, their physical disease burden is highly variable in terms of lesion number, size, location, and biology. Administration of the investigational agent must be feasible, reproducible, and well-tolerated by patients. Further, the personnel and setting of the injection must be considered—for palpable or superficial lesions this may be accomplished by a surgeon or oncologist in the clinic setting versus less discrete subcutaneous nodules that require assistance of a radiologist.

In early development, this population’s heterogeneity complicates the determination of classic trial measures such as maximum tolerated dose and dose-limiting toxicities. For instance, dose escalation may be accomplished by injecting different volumes of a fixed concentration, injecting a fixed volume with increasing concentration, or increasing both injection volume and concentration as a factor of lesion size [12]. In patients with multiple lesions, one or more may be injected. While the same amount of study agent may be administered at each escalation in these schemas, the bioavailability within an individual’s tumor microenvironment may vary. In addition, prior study of immunotherapeutic agents has shifted the priority of dose escalation trials. These agents often lack a linear dose-response relationship and dose-dependent toxicity; therefore, the focus has transitioned to the optimal biological dose (OBD) [10,12,53]. The OBD is often determined by looking at the tumor response itself, which requires pre-treatment and on-treatment biopsies of injected lesions. Further, some trials may include biopsies of non-injected lesions to determine distant effects. These biopsies provide significant pharmacodynamic information on these agents and their activation of the local tumor immune environment. Alternatively, pharmacokinetics may be gathered from peripheral blood and while not central to the development of intralesional therapies, may demonstrate the presence of systemic effects, if any, and possible contribution to noninjected lesions [12].

**Fig. 3.**
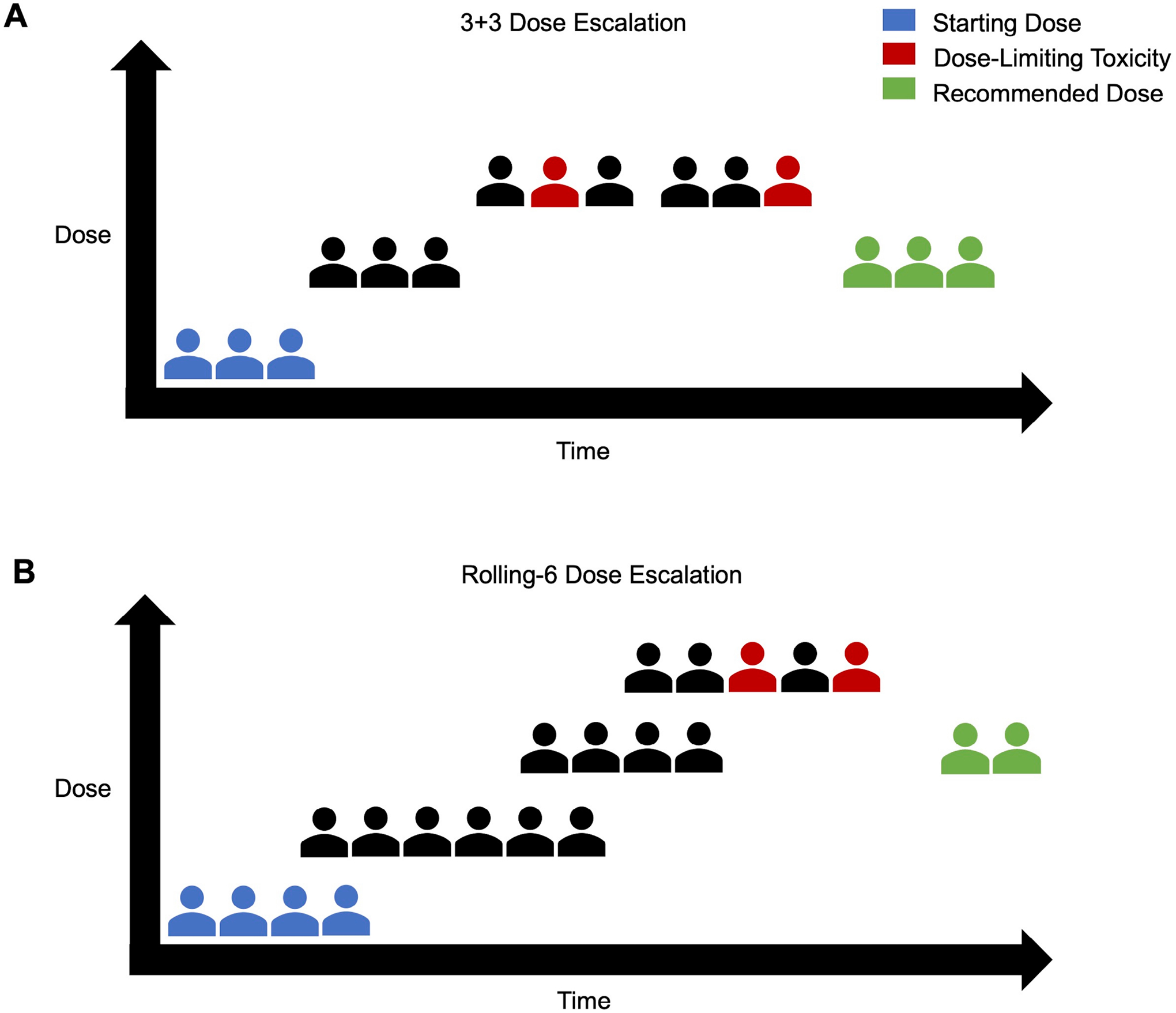
Dose Escalation Trial Schema. (A) Conventional 3+3 Design where 3 patients are started at the starting dose and sequential enrollment is done in groups of 3. If a dose-limiting toxicity is encountered, an additional 3 patients are enrolled at that same dose. If 2 or more experience a dose-limiting toxicity, the dose is reduced to the prior level. (B) Rolling-6 Design where 2–6 patients can be concurrently enrolled at the same dose level. De-escalation occurs similarly when 2 or more experience a dose-limiting toxicity; however, escalation can occur when 3/3, 4/4, 5/5, 5/6, or 6/6 patients do not experience a dose-limiting toxicity.

Altogether, these factors complicate early trial design, and some have suggested parting ways with the classic 3+3 design in favor of a rolling-six trial design (Fig. 3). This rolling-six design allows for enrollment of up to six patients at each dose, ultimately providing larger sample size and scientific data among this heterogeneous population [54,55]. Alternatively, including provisions to allow “backfilling” of patients to doses after they have been cleared for safety can strengthen the information gained from these trials. Marabelle and colleagues have published recommendations on inclusion/exclusion criteria for intralesional trials. They suggest that patients must have at least one tumor lesion amenable to injection, often defined as at least 1cm in diameter, although smaller diameter cutaneous or superficial lesions may be eligible [12,56]. Exclusion criteria include the use of anticoagulants (curative dose), prior significant bleeding diathesis, lesion proximity to large vascular structures or hollow organs, or thrombocytopenia (<50 k) [12,56]. Inclusion of patients on anti-platelet agents are at the discretion of the protocol, varying with lesion depth and location [56].

Perhaps the most challenging adaptation for intralesional trials, is the reliable determination of efficacy. Traditional oncology trials have relied on the well-defined Response Evaluation Criteria in Solid Tumours (RECIST); however, with the introduction of immunotherapies, the RECIST criteria did not account for features such as pseudoprogression, transient lymphadenopathy, nor the spectrum of mixed response [57,58]. Modified immune RECIST (iRECIST) are now utilized and intratumoral RECIST (itRECIST) criteria have been proposed [58,59]. The itRECIST criteria build upon historical standards with classification of measurable lesions into target and nontarget lesions. Both target and nontarget lesions are further defined as injected or noninjected lesions. While nontarget lesions cannot be reclassified as target lesions throughout the course of the study, noninjected lesions may become injected lesions [59]. Circumstances where this may be appropriate include those where noninjected lesions grow or injected lesions are no longer discernable. If biopsies are planned, they should be conducted on nontarget lesions only. Similar to standard RECIST, the imaging modality by which disease burden is measured should remain consistent throughout the trial. In addition to imaging, physical measurements (i.e., diameter) and characteristics of the lesions should be collected at each timepoint and injection. Overall response is assessed by changes in the sum of diameters (SOD) of all target lesions, qualitative changes in nontarget lesions, and accounts for any new lesions that appear [59]. Injected and noninjected responses are gathered from changes in SOD of target lesions in each respective category [56,59]. Disease progression is defined by iRECIST; however, mixed responses are not uncommon with some injected lesions regressing while noninjected lesions grow or new lesions appear [58,59]. In these cases, investigators may choose to switch injection strategy to include those new or enlarging lesions. Further, reassessment imaging may be completed at a wider interval (4–12 weeks) to provide time for treatment effect to manifest [59].

Trial design for oncolytic viruses requires creativity and attention to non-traditional details. Successful implementation necessitates a team of investigators, study coordinators, clinical nurses, and radiologists who are familiar with the aforementioned challenges. Ultimately, a well-designed intelligent protocol is essential as interpretation of these results will inform subsequent treatment regimens, injection intervals, and implementation into clinical practice.

## 6. Conclusions

Recurrent and advanced melanoma remain heterogeneous in presentation and disease burden. There are several treatment options available for this population ranging from surgical excision in cases of limited disease to systemic immune therapies for those with extensive disease. Despite the success of modern systemic therapy, many patients are non-responders or develop drug resistance. In this current landscape there is not an exact formula and treatment is certainly not “one size fits all”. Rather, treatment is multifaceted and should be personalized to each patient’s pattern of disease, performance status, and goals of care.

The superficial nature of IT melanoma and dermal metastases certainly make oncolytic viruses and other intralesional agents an attractive option, although the risk of developing systemic disease while on these agents must be considered. While recent literature suggests oncolytic viruses may not offer the systemic efficacy of modern immunotherapies or targeted therapy, either alone or in combination with systemic options, their safety profile is of less concern. In this setting, oncolytic viruses will likely continue to play a role in the management of patients with progressive disease on systemic therapy, those who choose to forego systemic therapy, or those with multiple comorbidities who are not able to tolerate systemic therapy. Further, local injection of oncolytic viruses may play a role in palliation of IT lesions, sparing patients morbid resection and optimizing quality of life.

At our institution, we take these considerations into account and employ a multidisciplinary approach on such cases. We believe that the complexity of treatment in recurrent and advanced melanoma requires a multidisciplinary team with engagement of medical oncologists, surgeons, radiologists, dermatologists, and clinical investigators. While there have been great advances in recent years, we continue to encounter challenging patterns of disease that do not respond to therapy. Ongoing trials may provide further information on the utility of oncolytic viruses in this setting; however, necessitate creativity in design and interpretation. At our institution, such trial development includes clinicians and scientists. Given the niche population in which these agents will likely be utilized, the definition of efficacy may continue to evolve. At this time, there is eager anticipation for trial results that may offer further perspective on the future of oncolytic viruses in melanoma.

## Abbreviations

DAMPs: damage-associated molecular patterns
DRR: Durable Response Rate
FDA: Food and Drug Administration
IT: In transit
ICI: immune checkpoint inhibitor
IFN: Interferon
OBD: Optimal Biologic Dose
ORR: Overall Response Rate
PFS: Progression Free Survival
RECIST: Response Evaluation Criteria in Solid Tumours
SOD: Sum of Diameters
T-VEC: talimogene laherparepvec
